# Single-nucleus transcriptome profiling unveils cell-type-specific ethylene and TOR signaling in tomato

**DOI:** 10.1093/hr/uhag044

**Published:** 2026-02-28

**Authors:** Wei Huang, Liujing Yang, Nan Hu, Qiu Jiang, Ping Zhou, Jing He, Li Lu, Zhong-hua Chen, Cong Tan

**Affiliations:** State Key Laboratory of Genome and Multi-omics Technologies, BGI Research, Wuhan 430047, China; Key Laboratory of Genomics, Ministry of Agriculture, BGI Bioverse, Shenzhen 518083, China; State Key Laboratory of Genome and Multi-omics Technologies, BGI Research, Wuhan 430047, China; College of Life Sciences, University of Chinese Academy of Sciences, Beijing 100049, China; College of Horticulture and Forestry, Tarim University, Alar, Xinjiang 843300, China; State Key Laboratory of Genome and Multi-omics Technologies, BGI Research, Wuhan 430047, China; School of Agriculture, Yunnan University, Kunming 650000, China; State Key Laboratory of Genome and Multi-omics Technologies, BGI Research, Wuhan 430047, China; School of Science, Western Sydney University, Penrith, NSW 2751, Australia; School of Pharmaceutical Sciences, Wuhan University, Wuhan 430071, China; School of Agriculture, Food and Wine, Waite Research Institute, Adelaide University, Glen Osmond, SA 5064, Australia; State Key Laboratory of Genome and Multi-omics Technologies, BGI Research, Wuhan 430047, China; Key Laboratory of Genomics, Ministry of Agriculture, BGI Bioverse, Shenzhen 518083, China

## Abstract

Plant etiolation, a critical process for seedling emergence, is regulated by ethylene and target of rapamycin (TOR) signaling pathways. However, the cell-type-specific regulation of these pathways remains poorly understood. To address this, we generated a comprehensive single-nucleus RNA transcriptome atlas of etiolated apical hooks and hypocotyls in tomato seedlings treated with the ethylene precursor aminocyclopropane-1-carboxylic acid (ACC), the TOR inhibitor Torin2, or a mock treatment. In total, we obtained high-quality gene expression profiles for 117 929 nuclei across these tissues and treatments. Our analysis identified seven major cell types within each tissue, revealing distinct cellular compositions and transcriptional programs. ACC treatment increased the proportion of epidermal cells in apical hooks, while Torin2 had limited impact on cellular composition. Differential gene expression analysis demonstrated tissue-specific sensitivity to these treatments: apical hooks exhibited extensive ACC-responsive differentially expressed genes, whereas hypocotyls were highly responsive to Torin2. Cellular responsiveness analysis uncovered divergent ethylene/auxin pathway activities, such as ACC-repressed auxin transport in hook endodermis-like cells. Dynamic trajectory analysis indicated both treatments altered cell differentiation, authenticating epidermis as the key cell type for ethylene-mediated etiolated growth. Crucially, we identified JA1 (HD-ZIP I TF) as a negative ethylene regulator enriched in epidermis, and CRISPR knockout *ja1* mutants exhibited hypersensitivity to ACC. This study deciphers cell-type-specific ethylene-TOR crosstalk, providing a robust single-cell RNA sequencing framework to dissect signaling networks in crops.

## Introduction

Development of plant seedling proceeds via two different programs in darkness and light, known as skotomorphogenesis and photomorphogenesis, respectively. After successful seed germination in soil, darkness acts as a signal for seedling etiolation, which is characterized by elongated hypocotyl, shortened root, and bended apical hook [1]. At this early stage of development, cell energy demand is high due to the heterotrophic growth. Etiolated growth is accomplished mainly by a complex network of transcription factors (TFs) like bHLH phytochrome interacting factors (PIFs) and phytohormones like auxin and ethylene [2–4]. The asymmetrical auxin gradient at the concave side and the convex side plays a predominant role in apical hook development [5]. Ethylene integrates with PIFs to promote skotomorphogenesis by regulating the expression of the acetyltransferase gene *HLS1*, leading to apical hook formation [6]. Furthermore, the target of rapamycin (TOR) kinase signaling pathway mediates hypocotyl elongation in both ethylene-dependent and independent manner. Inhibition of TOR triggers ethylene biosynthesis via the TAP46-ACS2/ACS6 link, resulting in repressed hypocotyl elongation [7]. EIN2, the central ethylene signaling regulon, acts as a direct substrate of glucose-activated TOR kinase in regulating hypocotyl elongation, and the regulation is independent of ethylene sensing and signaling [8]. In etiolated seedlings, TOR signaling integrates nutrient and energy availability to coordinate cell proliferation, organ growth, and metabolic reprogramming [9]. While both hormone and TOR signaling pathways have been extensively studied at the organismal or tissue level, their cell-type-specific regulatory mechanisms remain poorly resolved.

Traditional bulk RNA sequencing approaches obscure cellular heterogeneity. Recent advances in single-cell transcriptomics (scRNA-seq) have revolutionized the resolution of cellular diversity in plants, enabling the dissection of gene expression dynamics at unprecedented spatial granularity [10–13]. For instance, a time-series scRNA-seq analyses of the shoots and roots of de-etiolated *Arabidopsis* seedlings revealed the heterogeneity of light responses at cell-type resolution [14]. scRNA-seq analyses of healthy and ToCV-infected tomato leaves characterized a rapid function transition of mesophyll cells in virus-infected leaves [15]. Utilizing scRNA-seq in tomato identified that the suberized root exodermis is required for drought tolerance and that the shoot-borne roots initiate from phloem-associated cells through a unique transition state [16, 17]. These findings prompted us to unveil new insights into how distinct cell types differentially perceive and transduce ethylene or TOR signals in horticulturally important crops, especially under etiolation, a critical phase for seedling establishment.

Here, we constructed a comprehensive single-cell transcriptome atlas of etiolated tomato seedlings to decode cell-type-specific ethylene and TOR signaling architectures. By profiling 117 929 nuclei across hypocotyls and apical hooks, we identified transcriptionally distinct cell populations and mapped the spatiotemporal activity of ethylene-responsive and TOR-regulated genes. Our analysis revealed previously unrecognized crosstalk between ethylene and TOR signaling within specific cell types. These findings not only refine existing models of hormone signaling but also provide mechanistic insights into how cellular microenvironments modulate seedling morphogenesis. This work offers a framework for leveraging single-cell omics to dissect complex signaling networks in crops, with implications for improving stress resilience and growth efficiency in agricultural systems.

## Results

### SnRNA transcriptome profiling of etiolated apical hooks and hypocotyls

Germinated tomato (*Solanum lycopersicum*) seeds were cultivated in the dark for 72 h on 1/2 Murashige and Skoog (MS) medium. The medium was supplemented with either 1 μM 1-aminocyclopropane-1-carboxylic acid (ACC, a precursor of ethylene) or 5 μM Torin2 (a potent TOR kinase inhibitor), or received no treatment (Mock). Distinct tissue samples, including apical hooks and hypocotyls, were rapidly dissected and flash-frozen in liquid nitrogen to preserve transcriptional profiles. Nuclei isolation was performed using an optimized protocol, followed by library preparation for single-nucleus RNA (snRNA)-seq [18]. High-throughput sequencing on the MGI platform produced a total of 117 929 high-quality nuclei. The median values of unique molecular identifiers (UMIs) ranged from 991 to 1152, and the number of genes detected per nucleus ranged from 760 to 891. The total numbers of genes detected in the samples were as follows: apical hooks (Mock) 26 798, apical hooks (ACC) 28 490, apical hooks (Torin2) 29 827, hypocotyls (Mock) 28 704, hypocotyls (ACC) 30 895, and hypocotyls (Torin2) 29 509 (Table S1). Notably, to ensure the data reliability, we integrated raw data from at least two independent biological replicates for each sample (Fig. S1).

**Figure 1:**
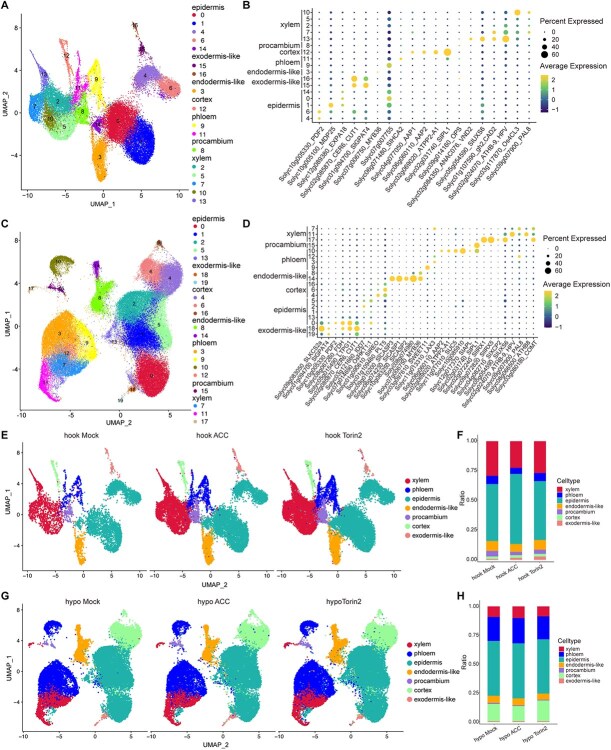
SnRNA-seq atlas of tomato etiolated apical hooks and hypocotyls treated with ACC or Torin2. (A) UMAP visualization of apical hook cells and the 17 cell clusters were further categorized into seven cell types. Each dot represents an individual nucleus. (B) UMAP visualization of hypocotyl cells and the 20 cell clusters were further categorized into seven cell types. Each dot represents an individual nucleus. (C and D) Dot plot depicts the top marker genes of distinct clusters. The dot size illustrates the percentage of cells expressing the gene. (E) UMAP visualization of predicted cell types from apical hooks under natural growth conditions (hook Mock), ACC treatment (hook ACC), and Torin2 treatment (hook Torin2). (F) Histogram showing proportions of cell types in hook Mock, hook ACC, and hook Torin2. (G) UMAP visualization of predicted cell types from hypocotyls under natural growth conditions (hypo Mock), ACC treatment (hypo ACC), and Torin2 treatment (hypo Torin2). (H) Histogram showing proportions of cell types in hypo Mock, hypo ACC, and hypo Torin2.

The transcriptome data of single-nucleus were subjected to Uniform Manifold Approximation and Projection (UMAP) for dimensionality reduction, resulting in 17 clusters for apical hook cells and 20 clusters for hypocotyl cells (Fig. 1A–D). To annotate cell types, we utilized previously reported well-known genes from tomato and homologous genes from *Arabidopsis* [14, 16, 17]. Both the 17 apical hook cell clusters and the 20 hypocotyl cell clusters can be broadly classified into seven major cell types: epidermis, exodermis-like, endodermis-like, cortex, phloem, procambium, and xylem.

In the hook samples, marker-based annotation assigned each cluster to a specific cell identity (Fig. 1A and B): clusters 0, 1, 4, 6, and 14 were defined as epidermis by the enrichment of the canonical epidermal genes *Solyc10g005330* (*PDF2*, HD-GL2 homeobox) [19], *Solyc10g005100* (*MDP25*, a calcium regulator of hypocotyl elongation) [20], and *Solyc12g089380* (*EXPA18*) [21]; clusters 15 and 16 were designated exodermis-like owing to the specific expression of *Solyc01g094700* (*SlGPAT4*) [22]; cluster 3 was identified as endodermis-like by its selective expression of *Solyc07g006750* (*MYB36*) [23], previously localized to endodermal cells by *in situ* hybridization; phloem identity was given to cluster 9 (*Solyc07g007755*) and cluster 11 (*Solyc06g071480*, *SIHCA2*, a nuclear DNA-binding protein confirmed in phloem by *in situ* hybridization) [24]; cluster 12 was labeled cortex based on specific enrichment of the cortex-expressed amino-acid transporter *Solyc04g077050* (*AAP1*) [25]; and clusters 2, 5, 7, 10, and 13 collectively formed the xylem group, jointly marked by *Solyc02g084350* (*ANAC076/VND2*) [26], *Solyc05g054590* (*SIUXS6*) [27], *Solyc01g107590* (*gh2/CAD2*), *Solyc02g024070* (*ATHB-9/HPV*) [28], *Solyc03g117870* (*Os4CL3*) [29], and *Solyc09g007900* (*PAL8*), with *UXS6* and *Os4CL3* already validated as xylem-specific through *in situ* hybridization and lignin staining, respectively.

For hypocotyl cells, the UMAP of cells and the specific expression of marker genes were shown in Fig. 1C and D. Clusters 0, 1, 2, 5, and 13 were annotated as epidermis according to the enriched expression of *Solyc10g005330* (*PDF2*) [19], *Solyc07g014690* (*HKT1;1*) [30], and *Solyc03g121660* (*IDD7*); *HKT1;1* lowers Na^+^ accumulation during salt stress and its GFP fusion localizes to epidermal cells [30]. Clusters 18 and 19 were defined as exodermis-like by high expression of *Solyc09g083050* (*SLKCS2a*) [16] and *Solyc01g094700* (*SlGPAT4*) [22]. Cortex was represented by clusters 4, 6, and 16, selectively expressing *Solyc01g090610* (*SIREO*) [31] and *Solyc01g109460* (*PLT5*); a SIREO-*uid*A reporter confirms cortex-specific activity in tomato roots [31]. Endodermis-like markers *Solyc09g010200* (*SICASP3*) [32], *Solyc06g074230* (*SICASP1*) [33, 34], *Solyc10g083250* (*SICASP2*) [32], and *Solyc07g006750* (*MYB36*) [23] were confined to clusters 8 and 14, and *pCASP1::CASP1-GFP* is activated exclusively in the endodermis-like [33]. Phloem was marked by *Solyc03g097610* (*SWEET11*) in cluster 9 and *Solyc10g085910* in cluster 10; *SWEET11* plasma-membrane localization in phloem has been demonstrated with *pAtSWEET::AtSWEET-GUS* staining [35]. Cluster 15 was designated procambium on the basis of specific expression of *Solyc02g031740* (*SlPL1*) [16] in the tomato single-cell atlas. Finally, xylem identity was given to clusters 7, 11, and 17, which collectively accumulate *Solyc02g072240* (*SIIRX1*) [36], *Solyc09g072820* (*SIIRX5*) [36], *Solyc04g082710* (*SIXCP2*) [37], and *Solyc05g054590* (*SIUXS6*) [27], while the xylem-associated homeodomain–leucine zipper gene *ATHB-8* promotes vascular differentiation in *Arabidopsis* [38]. Notably, ACC treatment in apical hooks resulted in a significant increase in the proportion of epidermis and exodermis-like cells, while the proportions of xylem, phloem, endodermis-like, and procambium cells decreased. ACC treatment in hypocotyls led to increased proportion of phloem cells while decreased proportion of endodermis-like cells. In contrast, Torin2 treatment had minimal impact on cellular composition in both apical hooks and hypocotyls (Fig. 1E–H).

### Identification of cell-type specific marker genes

**Figure 2:**
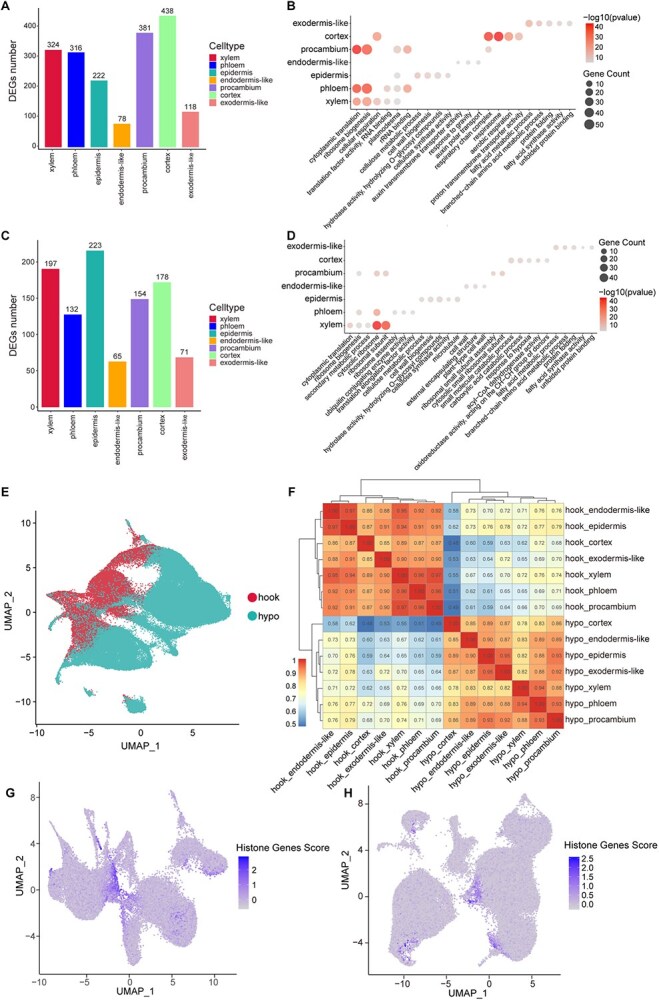
Features of different cell types in etiolated apical hooks and hypocotyls. (A) Numbers of characteristically upregulated DEGs in each of the seven apical hook cell types. (B) Numbers of characteristically upregulated DEGs in each of the seven hypocotyl cell types. (C) GO enrichment for the upregulated DEGs within each cell type of apical hooks. (D) GO enrichment for the upregulated DEGs within each cell type of hypocotyls. (E) UMAP visualization of total acquired nuclei from apical hooks and hypocotyls. (F) Heatmap showing correlations between different cell types from apical hooks and hypocotyls. (G) UMAP plots showing preferential expression of the histone-related genes in procambium cells of apical hooks. (H) UMAP plots showing preferential expression of the histone-related genes in epidermis cells of hypocotyls.

Among the seven apical hook cell types, we identified the highest number of upregulated differentially expressed genes (DEGs) in the cortex (438), followed by the procambium (381), xylem (324), phloem (316), epidermis (222), exodermis-like (118), and endodermis-like cells (78) (Fig. 2A; Table S2). In parallel, we found a greater number of upregulated DEGs in the epidermis (223), followed by the xylem (197), cortex (178), procambium (154), phloem (132), exodermis-like (71), and endodermis-like cells (65), across the seven hypocotyl cell types (Fig. 2B; Table S3). Except for some well-known marker genes, a large number of new marker genes have been identified, which are preferentially expressed in distinct cell types (Fig. S2).

To further validate the accuracy of cell type identification and to elucidate the molecular characteristics of each cell type, we performed gene ontology (GO) enrichment analysis on the upregulated DEGs (Fig. 2C and D). The upregulated genes in the apical hook and hypocotyl epidermis were enriched in pathways related to cell wall biogenesis and cellulose synthase/metabolic process. The upregulated genes in the apical hook and hypocotyl exodermis-like cells were enriched in pathways associated with protein folding and fatty acid synthase. The upregulated genes in the apical hook and hypocotyl phloem and xylem cells were enriched in pathways related to ribosome biogenesis. The upregulated genes in the apical hook procambium were enriched in pathways associated with cytoplasmic translation and ribosome biogenesis, while the upregulated genes in the hypocotyl procambium were enriched in pathways associated with cytosolic ribosome. The upregulated genes in the apical hook cortex were enriched in pathways related to the respirasome, whereas the upregulated genes in the hypocotyl cortex were enriched in pathways associated with small molecule/carboxylic acid catabolic processes. The upregulated genes in the apical hook endodermis-like cells were enriched in pathways related to auxin polar transport, while the upregulated genes in the hypocotyl endodermis-like cells were associated with cell wall pathways.

Considering the largely nonoverlapping feature between hypocotyl and apical hook cells (Fig. 2E), we further analyzed the correlations among different cell types of these two tissues. We discovered that the correlation scores between identical cell types in hypocotyls and apical hooks were even lower than the correlation scores between different cell types within the same tissue, indicating significant differences in cellular characteristics between the two tissues (Fig. 2F). For instance, we identified a set of histone-related genes exhibiting preferential expression in the procambium cells of apical hooks while not expressed in the procambium cells of hypocotyls (Fig. 2G and H), suggesting a more active cell division in apical hooks than that in hypocotyls.

### Analysis of ACC/Torin2-induced DEGs in different cell types

**Figure 3:**
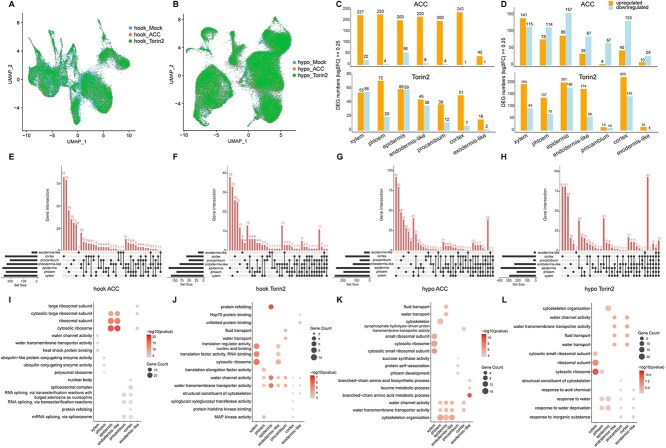
Cell-type-specific responses to ACC or Torin2 in etiolated apical hooks and hypocotyls. (A) UMAP visualization of apical hook cells under Mock, ACC treatment, or Torin2 treatment. (B) UMAP visualization of hypocotyl cells under Mock, ACC treatment, or Torin2 treatment. (C) Numbers of DEGs in seven apical hook cell types under ACC vs Mock and Torin2 vs Mock conditions. (D) Numbers of DEGs in seven hypocotyl cell types under ACC vs Mock and Torin2 vs Mock conditions. (E to H) Upset plot of DEGs among different cell types. (I to L) Dot plots of GO enrichment analysis of DEGs in each cell type.

To elucidate the specific changes in transcript abundance within individual cell-type clusters induced by ACC or Torin2 treatment, we conducted a differential analysis of single nucleus from seven distinct cell types by comparing samples from the Mock, ACC, and Torin2 treatments. The UMAP visualization of total cells from the Mock, ACC, and Torin2 samples revealed clear differences in cell numbers (Fig. 3A and B). In apical hooks, we identified 1456 and 529 DEGs across all seven cell types in response to ACC and Torin2 treatments, respectively (Fig. 3C; Table S4). In hypocotyls, we identified 1104 and 1517 DEGs across all seven cell types in response to ACC and Torin2 treatment, respectively (Fig. 3D; Table S5). Overall, the data indicate that a greater number of DEGs are involved in the ACC-treated apical hooks compared to the ACC-treated hypocotyls, while more DEGs were involved in the Torin2-treated hypocotyls than in the Torin2-treated apical hooks, demonstrating different sensitivities in the two tissues to ACC and Torin2 treatments (Fig. 3C and D). Notably, among the 1456 DEGs regulated by ACC in apical hooks, 1360 genes were upregulated. In contrast, a higher number of genes were downregulated among the ACC-regulated DEGs in hypocotyls (402 up vs 702 down) (Fig. 3C and D). For Torin2 treatment, 337 and 192 genes were upregulated and downregulated in apical hooks, respectively, while 958 and 559 genes were upregulated and downregulated in hypocotyls, respectively (Fig. 3C and D).

In apical hooks with ACC treatment, we identified the following numbers of DEGs in different cell types: 241 in cortex (240 up, 1 down), 228 in endodermis-like (220 up, 8 down), 259 in epidermis (203 up, 56 down), 41 in exodermis-like (40 up, 1 down), 234 in phloem (230 up, 4 down), 204 in procambium (200 up, 4 down), and 249 in xylem (227 up, 22 down) (Fig. 3C). In apical hooks with Torin2 treatment, we identified the following numbers of DEGs in different cell types: 58 in cortex (51 up, 7 down), 81 in endodermis-like (45 up, 36 down), 119 in epidermis (60 up, 59 down), 18 in exodermis-like (16 up, 2 down), 92 in phloem (72 up, 20 down), 50 in procambium (38 up, 12 down), and 111 in xylem (55 up, 56 down) (Fig. 3C). In hypocotyls with ACC treatment, we identified the following numbers of DEGs in different cell types: 178 in cortex (45 up, 133 down), 122 in endodermis-like (35 up, 87 down), 246 in epidermis (89 up, 157 down), 39 in exodermis-like (10 up, 29 down), 192 in phloem (78 up, 114 down), 71 in procambium (4 up, 67 down), and 256 in xylem (141 up, 115 down) (Fig. 3D). In hypocotyls with Torin2 treatment, we identified the following numbers of DEGs in different cell types: 366 in cortex (223 up, 143 down), 232 in endodermis-like (174 up, 58 down), 381 in epidermis (201 up, 180 down), 19 in exodermis-like (15 up, 4 down), 207 in phloem (137 up, 70 down), 24 in procambium (14 up, 10 down), and 288 in xylem (194 up, 94 down) (Fig. 3D; Tables S4 and S5).

### Discerning the cellular responses to ACC/Torin2 treatment

We conducted GO term enrichment analyses on these ACC/Torin2-induced DEGs. The 1456 DEGs in apical hooks with ACC treatment showed the enrichment of RNA splicing, ribosome biogenesis, and protein maturation pathway genes, while the 529 DEGs in apical hooks with Torin2 treatment displayed the enrichment of nucleoside triphosphate metabolic process, pyruvate metabolic process, and ribosomal large subunit biogenesis pathway genes (Fig. S3A and B). The 1104 DEGs in hypocotyls with ACC treatment were enriched in pathways associated with water deprivation response, salt response, and fatty acid metabolic process pathway genes, while the 1517 DEGs in hypocotyls with Torin2 treatment were enriched in pathways related to ADP metabolic process, glycolytic process, and protein-containing complex disassembly (Fig. S3C and D).

To identify potential candidate genes that regulate the cellular response of etiolated apical hooks and hypocotyls to ACC and Torin2 treatments, we further conducted multiple pairwise comparisons of DEGs among various cell types. The Venn diagram illustrating DEGs across the seven cell types revealed that 18, 4, 8, and 13 overlapping DEGs were identified in the comparisons of hook-ACC vs hook-Mock, hook-Torin2 vs hook-Mock, Hypo-ACC vs Hypo-Mock, and Hypo-Torin2 vs Hypo-Mock, respectively (Fig. S4; Table S6). Interestingly, *Solyc00g500164* was characterized as the only gene that was downregulated in all seven cell types across the two tissues in response to ACC and Torin2 treatments. *Solyc00g500164* is a functionally unknown gene, and its homologs can only be found in the tomato wild relative species *S. chilense*, which is well known for the remarkable resilience against biotic and abiotic stresses [39]. In ACC-treated apical hooks, 82, 35, 79, 1, 45, 31, and 29 DEGs were specifically presented in cortex, endodermis-like, epidermis, exodermis-like, phloem, procambium, and xylem, respectively (Fig. 3E; Table S6). In Torin2-treated apical hooks, 26, 12, 38, 6, 25, 4, and 33 DEGs were specifically presented in cortex, endodermis-like, epidermis, exodermis-like, phloem, procambium, and xylem, respectively (Fig. 3F; Table S6). In ACC-treated hypocotyls, 39, 12, 80, 24, 28, 48, and 92 DEGs were specifically presented in cortex, endodermis-like, epidermis, exodermis-like, phloem, procambium, and xylem, respectively (Fig. 3G; Table S6). In Torin2-treated hypocotyls, 81, 16, 81, 1, 9, 0, and 69 DEGs were specifically presented in cortex, endodermis-like, epidermis, exodermis-like, phloem, procambium, and xylem, respectively (Fig. 3H; Table S6). In addition, we identified DEGs of individual cell type that are specifically or commonly regulated by ACC and/or Torin2 treatments in the two tissues (Fig. S5). For instance, we found that the majority of upregulated DEGs (46/60 DEGs) in the Torin2-treated apical hook epidermis are among the upregulated DEGs in the ACC-treated apical hook epidermis (Fig. S5). Notably, most of the DEGs were cell-specific, indicating that different cell types had unique responses to ACC or Torin2 treatment. In line with that, the GO enrichment analysis revealed enrichment of different pathways underlying these DEGs in each cell type (Fig. 3I–L).

**Figure 4:**
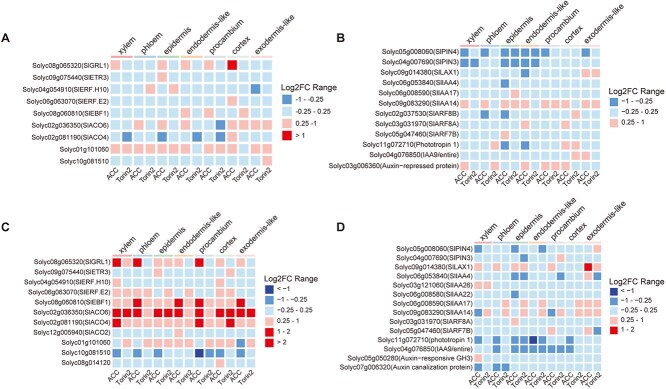
Expression patterns of ethylene and auxin pathway genes upon ACC/Torin2 treatments. (A) Heatmap showing relative expression levels of nine ethylene pathway-associated genes in seven apical hook cell types under ACC vs Mock and Torin2 vs Mock conditions. (B) Heatmap showing relative expression levels of 12 auxin pathway-associated genes in seven apical hook cell types under ACC vs Mock and Torin2 vs Mock conditions. (C) Heatmap showing relative expression levels of 11 ethylene pathway-associated genes in seven hypocotyl cell types under ACC vs Mock and Torin2 vs Mock conditions. (D) Heatmap showing relative expression levels of 14 auxin pathway-associated genes in seven hypocotyl cell types under ACC vs Mock and Torin2 vs Mock conditions.

Particularly, we found a set of genes associated with ethylene and auxin pathways among these DEGs. *Solyc01g101060*, which encodes an *S*-adenosyl-l-methionine synthetase involved in ethylene biosynthesis, was significantly upregulated in five and seven cell types of apical hooks following ACC and Torin2 treatments, respectively (Fig. 4A). *SlIAA14* (*Solyc09g083290*) was significantly induced in seven and three cell types of apical hooks by ACC and Torin2 treatments, respectively (Fig. 4B). Two auxin efflux genes, *SlPIN3* (*Solyc04g007690*) and *SlPIN4* (*Solyc05g008060*), were significantly downregulated in the epidermis and endodermis-like cells of apical hooks in response to ACC and Torin2 treatments (Fig. 4B), indicating the involvement of auxin signaling at the downstream of ethylene and TOR signaling during apical hook development. *SlACO6* (*Solyc02g036350*), which encodes a 1-aminocyclopropane-1-carboxylate oxidase of ethylene biosynthesis, was significantly upregulated by both ACC and Torin2 treatments in all seven cell types of hypocotyls (Fig. 4C). Two other genes related to ethylene biosynthesis and signaling, *SlACO4* (*Solyc02g081190*) and *SlEBF1* (*Solyc12g009560*), exhibited higher expression in specific cell types in response to ACC and Torin2 of hypocotyls (Fig. 4C). The auxin influx gene *SlLAX1* (*Solyc09g014380*) was downregulated by ACC in the endodermis-like cells of apical hooks, while it was induced by ACC in the xylem, phloem, epidermis, procambium, and exodermis-like cells of hypocotyls (Fig. 4B and D). *Phytotropin 1* (*Solyc11g072710*) and *IAA9*/*entire* (*Solyc04g076850*) were significantly downregulated in the epidermis and endodermis-like cells of hypocotyls in response to ACC and Torin2 treatments (Fig. 4D). These data reveal both distinct response and common strategies of ethylene and TOR signaling at the cellular level during plant etiolation.

To further explore the diversity of ethylene, auxin, and TOR signaling at the cellular level, we analyzed the expression scores of 66 genes from the ethylene pathway, 36 genes from the TOR pathway, and 236 genes from the auxin pathway (Table S7). Following treatment with ACC or Torin2, the TOR pathway genes showed significantly higher expression scores in all seven cell types of apical hooks, while the auxin pathway genes demonstrated altered expression in specific cell types, such as the epidermis and phloem cells of the apical hooks (Fig. S6A); the genes associated with the ethylene pathway exhibited significantly higher expression scores across all seven cell types in hypocotyls (Fig. S6B). Notably, ACC treatment resulted in lower expression for auxin pathway genes in the epidermis and endodermis-like of the apical hooks, whereas Torin2 treatment led to higher expression for auxin pathway genes in the xylem, phloem, epidermis, and cortex cells of the apical hooks (Fig. S6A). In hypocotyls, ACC treatment caused a decrease in the expression of auxin pathway genes in the phloem and cortex cells, while Torin2 treatment resulted in increased expression for these genes in the phloem and cortex cells (Fig. S6B). Additionally, ACC treatment led to lower expression for auxin pathway genes in the epidermis and endodermis-like cells, while higher expression scores were observed in the procambium- and exodermis-like cells of ACC-treated hypocotyls (Fig. S6B).

### Altered cell differentiation trajectory and capacity upon ACC/Torin2 treatment

**Figure 5:**
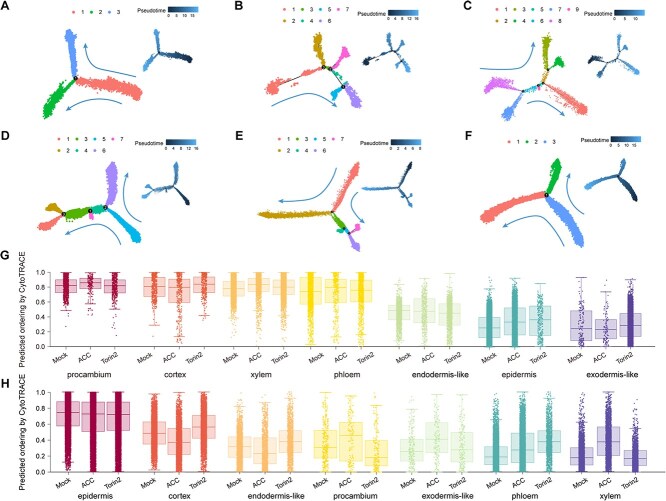
ACC/Torin2 treatments altered cell fate determination programs in etiolated apical hooks and hypocotyls. (A to C) Distribution of seven apical hook cells along the pseudo-time trajectory branches under Mock (A), ACC treatment (B), and Torin2 treatment (C). (D to F) Distribution of seven hypocotyl cells along the pseudo-time trajectory branches under Mock (D), ACC treatment (E), and Torin2 treatment (F). (G) Cell differentiation ability score of seven apical hook cells under Mock, ACC treatment, or Torin2 treatment. The degree of cell differentiation gradually increases from left to right. (H) Cell differentiation ability score of seven hypocotyl cells under Mock, ACC treatment, or Torin2 treatment. The degree of cell differentiation gradually increases from left to right.

To evaluate the effects of ACC and Torin2 treatments on cell differentiation during skotomorphogenesis, we conducted pseudo-time analysis using Monocle to confirm their developmental trajectories. In apical hooks, both ACC and Torin2 treatments resulted in an increased number of branches and states (Fig. 5A–C), indicating that both ACC and Torin2 treatments significantly influenced the differentiation process of etiolated seedlings. Next, the changes in gene expression were categorized according to trends observed over pseudo-time and across different branches. Four gene clusters were identified for each of the three samples of apical hooks (Fig. S7A–C). Similar expression trends were noted between the Torin2-treated and mock samples, while the ACC-treated sample exhibited a markedly different expression pattern compared to the other two samples (Fig. 5A–C). In hypocotyls, only Torin2 treatment led to a decrease in branch and state numbers (Fig. 5D–F). Likewise, four gene clusters were obtained for each of the three samples of hypocotyls (Fig. S7D–F).

We predicted the relative differentiation status of cells by detecting reductions in transcriptional diversity as markers of pseudo-frequency projections along trajectories. This approach allows us to estimate the cells’ capacity to differentiate [40]. We found that the procambium and epidermis exhibit the highest differentiative capacities in apical hooks and hypocotyls, respectively (Fig. 5H and I). In apical hooks, treatment with ACC increased the differentiative capacity of procambium, xylem, phloem, and epidermis cells, while treatment with Torin2 enhanced the differentiative capacity of cortex, xylem, phloem, epidermis, and exodermis-like cells (Fig. 5H). We further analyzed the gene modules associated with the epidermis due to its enhanced differentiative capacity following ACC and Torin2 treatments, and the GO term analysis revealed significant enrichment of distinct pathways across the three samples (Fig. S8A and B). In hypocotyls, ACC treatment inhibited the differentiative capacity of cortex and endodermis-like cells, while it increased the differentiative capacity of procambium, exodermis-like, phloem, and xylem cells. Torin2 treatment inhibited the differentiative capacity of procambium cells, while it increased the differentiative capacity of cortex, endodermis-like, exodermis-like, and phloem cells (Fig. 5I). Likewise, we further analyzed the gene modules associated with epidermis in the three hypocotyl samples, and the GO term analysis showed the enrichment of distinct pathways (Fig. S8C and D). Notably, cell wall-related pathway genes were highly enriched in both ACC-treated apical hook epidermis and ACC-treated hypocotyl epidermis (Fig. S8B and D). These results suggest intrinsic differences in the differentiative capacity of the two tissues and reveal that cell wall remodeling in epidermis might be a key biological program in ethylene-mediated etiolated seedling growth.

### Differentiation trajectory of epidermis affected by ACC and Torin2 treatments

**Figure 6:**
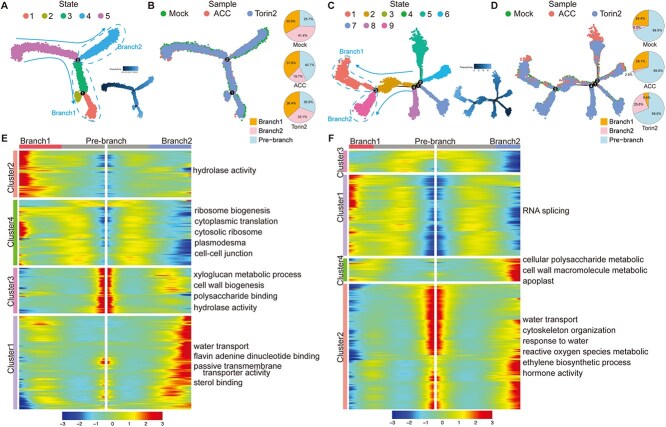
Differentiation trajectory of epidermis cells. (A) Pseudo-time trajectory analysis of epidermis of apical hooks. (B) Distribution difference of cells along the pseudo-time trajectory of epidermis of apical hooks. (C) Pseudo-time trajectory analysis of epidermis of hypocotyls. (D) Distribution difference of cells along the pseudo-time trajectory of epidermis of hypocotyls. (E and F) Heatmap showing the expression of regulatory genes on pseudo-time trajectories of epidermis of apical hooks and hypocotyls, and the GO enrichment analyses of each gene cluster were shown on the right.

It has been documented that epidermis as the key cell type required for ethylene-mediated hypocotyl and apical hook development [41, 42]. To examine if the physiological states of epidermis of etiolated seedlings were rewired by ACC or Torin2 treatment, the gene expression matrices of the epidermis generated from apical hook and hypocotyl samples were used to construct the differentiation trajectory (Fig. 6A–D). The pseudo-time trajectory analysis results revealed clear bifurcation points that divided epidermis cells into five and nine states in apical hooks and hypocotyls, respectively (Fig. 6A and C). The trajectory diverted into two main branches for both apical hook and hypocotyl epidermis. Intriguingly, we found that cells from the mock and treatment groups were unequally distributed along the two branches, suggesting dramatic but heterogeneous rewiring of the epidermis transcriptome under ACC and Torin2 treatments (Fig. 6B and D).

To uncover key genes and pathways that might mediate the state transition of epidermis cells, we screened the DGEs along the pseudo-time line and performed cluster analysis. Four hundred three and 582 DEGs were identified across the pseudo-time order of apical hook and hypocotyl epidermis cells, respectively (Fig. 6E and F; Table S8). Based on the similarity of expression trends, these DEGs were further divided into four clusters that showed distinct gene expression patterns, representing a transcriptional rewiring program in epidermis development. The GO analysis revealed the enrichment of different pathways along differentiation stages (Fig. 6E and F). Collectively, these results suggest the fundamental roles of ethylene and TOR signaling-mediated cellular differentiation of epidermis during skotomorphogenesis.

### Coexpression modules of DEGs in ethylene and TOR signaling-mediated etiolated growth at single-cell level

**Figure 7:**
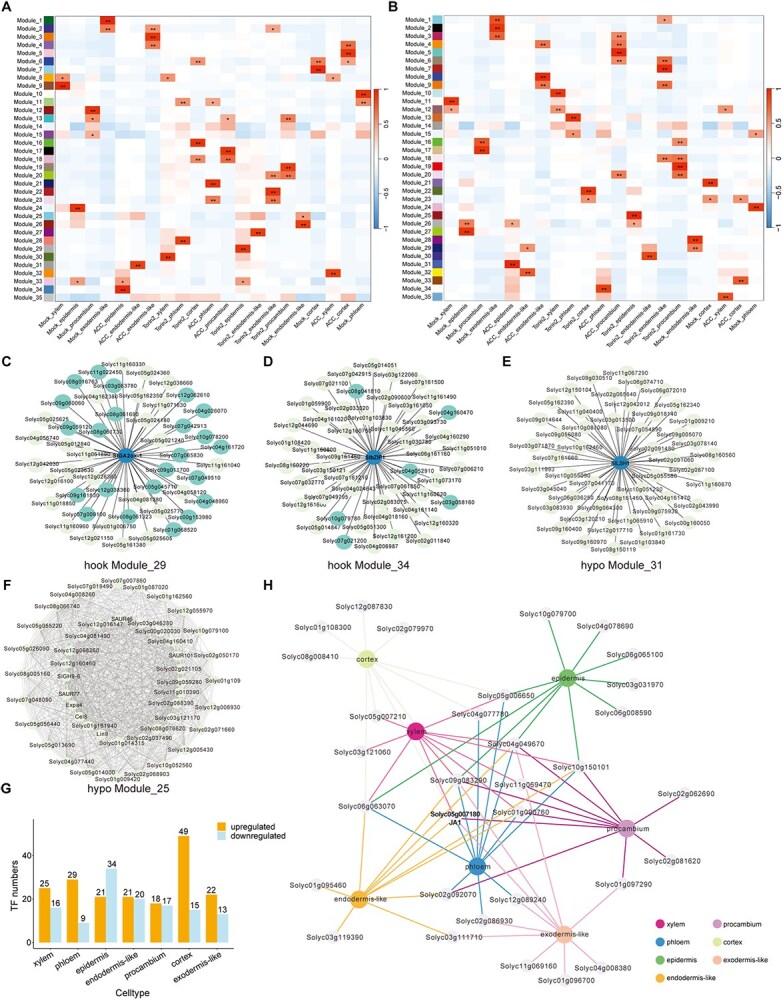
Coexpression correlation modules with key DEGs in seven cell types in response to ACC or Torin2. (A) Thirty-five coexpression modules associated with each cell type of apical hooks under ACC and Torin2 treatments. (B) Thirty-five coexpression modules associated with each cell type of hypocotyls under ACC and Torin2 treatments. (C to F) Four gene association modules harboring key DEGs in response to ACC or Torin2 treatment. (G) Numbers of differentially expressed TF genes in each of the seven cell types in response to ACC and Torin2 treatments. (H) Top 10 representative TF regulons for each cell type.

To reveal dynamic patterns and the single-cell transcriptional regulatory networks of ethylene and TOR signaling in tomato apical hooks and hypocotyls, a gene coexpression network analysis of genes across all seven cell types was performed using WGCNA analysis. For both apical hooks and hypocotyls, each resultant coexpression network was composed of 35 modules (Fig. 7A and B; Table S9). The vast majority of these modules exhibited significant correlations with at least one population. Eighteen, 16, and 16 modules were associated with mock, ACC, and Torin2 populations of apical hooks, respectively (Fig. 7A). Fifteen, 16, and 17 modules were associated with mock, ACC, and Torin2 populations of hypocotyls, respectively (Fig. 7B). We further focused on modules corresponding to the ACC- and Torin2-treated epidermis, including modules 29 and 34 of apical hooks and modules 25 and 31 of hypocotyls (Fig. 7C–F). Analyzing genes in these modules identified *SlGA2ox-1* (*Solyc05g053340*), encoding a metabolic enzyme of gibberellin, as a central regulator linked to Torin2-treated epidermis of apical hooks (Fig. 7C), the TF gene *SlbZIP1* (*Solyc01g079480*) as a central regulator linked to ACC-treated epidermis of apical hooks (Fig. 7D), the TF gene *SlLSH1* (*Solyc04g009980*) as a central regulator linked to ACC-treated epidermis of hypocotyls (Fig. 7E), and several auxin-related *SAUR* genes as well as cell wall remodeling genes as key regulators for the Torin2-treated epidermis of hypocotyls (Fig. 7F). The involvement of auxin, gibberellin, and cell wall-related genes indicates a complicated regulatory axis and phytohormone cross-talk underlying the ethylene and TOR signaling-mediated etiolated growth at single-cell level.

### JA1 as a core regulator in ethylene-mediated etiolated growth

To investigate the potential TFs involved in ethylene and TOR signaling in etiolated hypocotyls and apical hooks, we screened the differentially expressed TF genes across all the seven cell types. Treatment with ACC or Torin2 altered the expression of 86 TF genes, with the majority found in the cortex (49 up, 15 down), followed by the epidermis (21 up, 34 down) (Fig. 7G). These TF genes belong to several families, including bHLH, HD-ZIP, MADS-box, zinc finger, ARF, IAA, and ERF, among others, exhibiting both cell type- and tissue-specific responses to ACC and Torin2 treatments (Fig. S9A; Table S10). We further analyzed the top 10 representative TFs for each cell type and found an auxin-signaling repressor gene, *SlIAA14* (*Solyc05g083290*), as the sole TF that was dysregulated in all seven cell types (Fig. 7H). Consistently, the protein interaction network analysis of these TFs suggests that auxin signaling may serve as the core effector in response to ethylene and TOR signaling (Fig. S9B). In addition, we found that a homeodomain leucine zipper I (HD-ZIP I) TF family gene, *JA1* (*Solyc05g007180*), was dysregulated in six cell types except in epidermis (Fig. 7H), implying its high potential but uncharacterized role in etiolated seedling growth.

**Figure 8:**
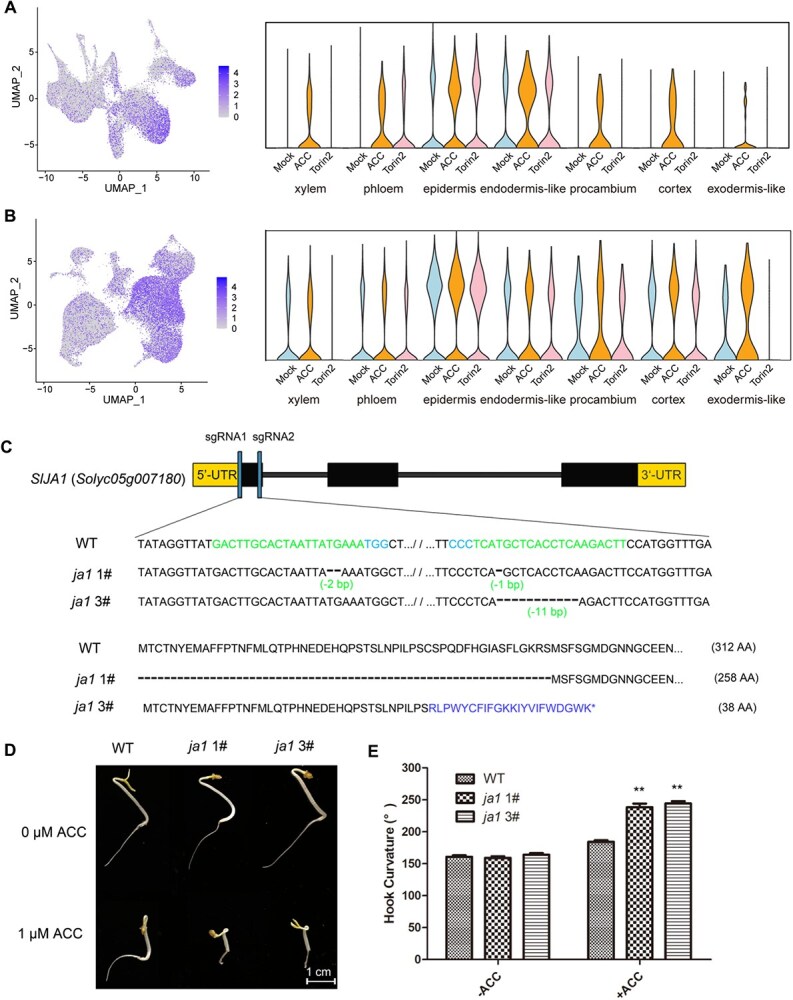
Characterization of JA1 as a key TF in modulating ethylene-mediated skotomorphogenesis. (A) UMAP plot and violin plot exhibiting the expression of *JA1* in each of the seven apical hook cell types responding to ACC or Torin2 treatment. (B) UMAP plot and violin plot exhibiting the expression of *JA1* in each of the seven hypocotyl cell types responding to ACC or Torin2 treatment. (C) Generation of two mutant alleles of *JA1* through the CRISPR/Cas9 system. (D) Ethylene triple response assay of WT and two *ja1* mutant alleles. (E) Statistics of apical hook curvature of WT and two *ja1* mutant alleles in (D). The error bars indicate the SDs (*n* = 10). ^**^P < 0.01, Student’s *t* test.

As the role of auxin in apical hook and hypocotyl development has been intensively studied, we chose JA1 as the candidate to further explore its function during etiolated growth. Notably, the *JA1* homolog *AtHB13* is involved in sugar signaling and acts as a cell division regulator in *Arabidopsis* [43]. Although *JA1* is preferentially expressed in the epidermis of both apical hooks and hypocotyls, it was significantly induced by ACC treatment in six cell types of apical hooks and in the exodermis-like cells of hypocotyls (Fig. 8A and B), suggesting that ethylene might be required for the expression patterns of *JA1*. Therefore, we applied the CRISPR-Cas9 system to generate loss-of-function alleles of *JA1* in tomato, resulting in two alleles with different genome-editing events (Fig. 8C). Importantly, the etiolated seedlings of *ja1* mutants were comparable with WT etiolated seedlings under normal condition but exhibited hypersensitivity to ACC treatment, with exaggerated hook angle and shortened hypocotyl and root length (Fig. 8D and E; Fig. S10), suggesting a negative role for *JA1* in ethylene-mediated etiolated growth during skotomorphogenesis.

## Discussion

### Tissue-specific cell-type composition and transcriptional landscapes

Our snRNA-seq analysis of etiolated tomato hypocotyls and apical hooks under ACC and Torin2 treatments revealed profound cell type-specific transcriptional reprogramming. Despite the shared annotation of seven major cell types (epidermis, exodermis-like, endodermis-like, cortex, phloem, procambium, and xylem) in both tissues, their transcriptomic profiles exhibited remarkably low correlations (Fig. 2F), underscoring intrinsic differences in cellular identity and functional plasticity of plant cells across tissues. This divergence was further emphasized by the distinct GO enrichment patterns of cell type-specific genes (Fig. 2C and D). For instance, totally different GO terms were identified in genes preferentially expressed in endodermis-like cells of hypocotyls and apical hooks; the hypocotyl epidermal cells showed enrichment in ‘microtubule binding’ pathway genes, which were not found in the apical hook epidermis cells. In contrast, some xylem preferentially expressed genes in apical hooks were associated with ‘proton transmembrane transport’, which were not found in hypocotyl xylem cells.

In addition, the snRNA-seq analysis uncovered striking differences in cell-type proportions between apical hooks and hypocotyls under ACC and Torin2 treatments (Fig. 1F and H). The ACC-induced increase in epidermis cell proportion in apical hooks, coupled with reductions in phloem and endodermis-like cells, aligns with ethylene’s established role in promoting apical hook formation and curvature through asymmetric cell elongation. Ethylene is known to suppress auxin transport in the hook region, creating auxin gradients that drive differential cell expansion [44]. The preferential upregulation of epidermal cell wall biogenesis genes (e.g. cellulose synthases) in ACC-treated apical hooks further supports this model, as cellulose deposition is critical for directional growth [45, 46]. Conversely, the limited alteration in cell-type proportions under Torin2 treatment suggests that TOR signaling may predominantly regulate metabolic and translational processes rather than cell fate determination in these tissues.

### Ethylene and TOR signaling at cellular resolution

In apical hooks, ACC triggered a robust transcriptional response (1456 DEGs, predominantly upregulated), whereas hypocotyls showed fewer DEGs (1104) with a bias toward downregulation (Fig. 3C and D). Conversely, Torin2 induced more DEGs in hypocotyls (1517) than in apical hooks (529) (Fig. 3C and D). This tissue specificity likely stems from their distinct developmental priorities: apical hooks rely heavily on ethylene-mediated differential growth for soil emergence [47], while hypocotyls prioritize elongation through TOR-driven cell expansion [8]. The dominance of upregulated DEGs in ACC-treated apical hooks suggests ethylene acts as a potent activator of transcriptional programs in this tissue.

ACC treatment elicited a pronounced upregulation of the ethylene biosynthetic genes and the ethylene signaling genes, including *SlACO4*, *SlACO6*, *SlGRL1*, and *SlEBF1*, across all seven cell types of hypocotyls (Fig. 4C). The systemic induction of these genes suggests a feedforward loop amplifying ethylene production, consistent with the hormone’s autoregulatory nature [48]. Intriguingly, ACC suppressed auxin transporting genes *SlPIN3* and *SlPIN4* in apical hook epidermis and endodermis-like cells (Fig. 4B), corroborating the ethylene-auxin crosstalk observed in *Arabidopsis* hook development [49]. The downregulation of auxin efflux carriers likely disrupts polar auxin transport in the apical hook, reinforcing asymmetric growth. In hypocotyls, however, ACC induced the auxin efflux carrier *SlLAX1* in xylem, phloem, epidermis, procambium, and exodermis-like cells (Fig. 4D), implicating *SlLAX1* as a hub gene for ethylene–auxin interplay during hypocotyl elongation.

Notably, the enrichment of ribosome biogenesis genes in phloem and xylem cells (Fig. 2C and D) aligns with TOR’s canonical role in promoting anabolic processes under energy-replete conditions [50]. Torin2’s induction of auxin pathway genes in apical hook xylem and cortex cells (Fig. S6A) suggests a compensatory mechanism to sustain growth under TOR inhibition, possibly via enhanced auxin-mediated cell expansion [44]. Torin2 treatment triggered distinct transcriptional responses, with hypocotyls exhibiting greater sensitivity than apical hooks (Fig. 3C and D). In certain cell types, the contrasting effects of ACC and Torin2 on ethylene, TOR, and auxin pathway-related genes highlight the nuanced interplay between ethylene, TOR, and auxin in balancing growth and stress adaptation (Fig. S6A and B).

### Cell type-specific differentiation trajectories

Pseudo-time analysis uncovered distinct effects of ACC and Torin2 on differentiation trajectories. In apical hooks, both treatments increased branch and state numbers, implying heightened cellular plasticity (Fig. 5A–C). This aligns with the ACC-driven enrichment of histone-related genes in procambium cells (Fig. 2G), which may prime cells for division or fate transitions. Conversely, hypocotyls exhibited reduced differentiation complexity under Torin2 (Fig. 5D–F), likely reflecting a trade-off between elongation and differentiation. Torin2 treatment exhibited severe inhibition of cell differentiation in hypocotyl, suggesting a more important role of TOR signaling in cell fate determination than that of ethylene signaling, which is consistent with the fact that TOR signaling is intimately connected with the nutrient assimilation and energy consumption in heterotrophic skotomorphogenesis [51]. CytoTRACE analysis further highlighted tissue-specific differentiation capacity: ACC enhanced the differentiative capacity of apical hook procambium and epidermis cells while suppressing hypocotyl cortex and endodermis, whereas Torin2 promoted differentiation in apical hook cortex, phloem, and epidermis while suppressing hypocotyl procambium (Fig. 5G and H). These results suggest that ethylene prioritizes apical hook morphogenesis by maintaining stem cell-like populations, while TOR sustains hypocotyl elongation via translational machinery.

### Transcription factor networks and JA1 as a key regulator

Upon ACC/Torin2 treatments, the identification of 86 differentially expressed TFs underscores the complexity of ethylene/TOR signaling integration (Fig. 7G). The protein interaction network’s centrality of auxin-related TFs (e.g. ARFs, IAAs) supports auxin signaling as a convergence point for ethylene and TOR pathways (Fig. S9B). Notably, JA1, an HD-ZIP I TF, emerged as a critical node in ethylene signaling. The ACC-induced upregulation of *JA1* and the hyper-acceleration of hook curvature in *ja1* mutants under ACC treatment (Fig. 8A–E) positions JA1 as a negative regulator of ethylene responses, potentially by dampening auxin signaling and cell division.

In conclusion, by dissecting the single-cell transcriptomic landscapes of ethylene- and TOR-modulated hypocotyls and apical hooks, this study unveils tissue- and cell type-specific regulatory networks governing skotomorphogenesis. The divergent responsiveness of these tissues highlights the contextual nature of hormone signaling, while the identification of JA1 underscores the complexity of transcriptional integration and advances the mechanistic understanding of apical hook and hypocotyl development. These insights lay the groundwork for manipulating ethylene–TOR interplay to optimize seedling establishment in agronomically challenging environments.

## Methods

### Plant material and sample preparation

To generate a comprehensive snRNA transcriptome atlas of etiolated apical hooks and hypocotyls in tomato, *S. lycopersicum* cv. Micro-Tom served as the wild type. Seeds were surface-sterilized with 70% ethanol for 3 min, then in 4% sodium hypochlorite for 18 min. After several washes with sterile distilled water, they were germinated in sterile tissue-culture flasks containing distilled water for 48 h in darkness. Uniformly germinated seeds were transferred to ½-strength Murashige and Skoog (MS) medium solidified without hormones and supplemented with 1 μM ACC, 5 μM Torin2, or no additives (Mock), respectively. Following 72 h of growth at 25°C in darkness, apical hooks and hypocotyls of etiolated seedlings were dissected with scalpels, snap-frozen in liquid nitrogen, and stored at −80°C for subsequent nuclei isolation.

### Single nuclei isolation

Single nuclei were isolated for six samples, including hook and hypocotyls tissues of plant treated with ACC, Torin2, and control. The experiment for single nuclei isolation is similar to that described in the complete single-nuclei transcriptome atlas of *Arabidopsis* [18], and brief steps were given below. Approximately, 0.2 g of freshly harvested tissue was snap-frozen in liquid nitrogen and homogenized in 3 ml of chilled NIBA buffer (0.8 M sucrose, 10 mM MgCl₂, 25 mM Tris–HCl pH 8.0, 0.1 mM DTT, 0.4 U ml ^− 1^ RNase inhibitor, 0.1 mM PMSF) at 4°C. The slurry was filtered sequentially through 40 and 30-μm cell strainers, then incubated on ice for 10 min to release nuclei. After a low-speed spin (50 g, 5 min, 4°C) to remove debris, the supernatant was centrifuged at 2000 *g* for 10 min to pellet nuclei. The pellet was gently resuspended in 1-ml Buffer B (0.4 M sucrose, 10 mM MgCl₂, 25 mM Tris–HCl, pH 8.0, 1% Triton X-100, 0.2 U μl^−1^ RNase inhibitor, 0.1 mM PMSF) and layered onto a discontinuous Percoll gradient (25%/75%). Following centrifugation at 3000 *g* for 15 min, the nuclei-enriched interface was collected, washed twice with Buffer B, and finally resuspended in cell resuspension buffer. Nuclear integrity and concentration were assessed by DAPI staining and hemocytometry, yielding ≥90% intact nuclei with minimal cytoplasmic contamination, ready for downstream snRNA-seq library construction.

### SnRNA-seq and data processing

snRNA sequencing libraries were generated on the BGI DNBelab C4 platform following the manufacturer’s protocol for the C Series scRNA Preparation Kit (MGI, 1000021082). After density-gradient isolation, nuclei were counted and diluted to 1000 nuclei μl ^− 1^ in ice-cold resuspension buffer. After RT, beads were treated with 200-μl exonuclease mix (37°C, 45 min) to remove unincorporated primers, followed by 13 cycles of PCR amplification (98°C 20 s, 58°C 20 s, 72°C 3 min). Amplified cDNA was purified with 60-μl AMPure XP beads, quantified by Qubit dsDNA HS assay, and sheared to 250- to 400-bp fragments using NEBNext dsDNA Fragmentase. Final libraries were constructed and sequenced on a BGISEQ-T7RS instrument (41-bp read 1, 100-bp read 2, 10-bp index).

The raw reads were aligned to the *S. lycopersicum* reference genome (v4.0) by STAR (v2.7.4a) algorithm. After constructing the gene expression matrix, data were processed by filtering low-quality and invalid cells by DoubletFinder (v2.0.3) for the subsequent analysis [52]. The matrix of cell-gene UMI count was then obtained from PISA after the contamination fractions were filtered using SoupX (v1.4.8).

### Quality control and unsupervised clustering

After generating the cell–gene count matrix, rigorous preprocessing was applied to ensure the reliability of downstream analyses. Nuclear selection was performed through two sequential filtering steps [18]. First, an initial filter removed nuclei with fewer than 200 detected genes as well as genes detected in fewer than three nuclei. Subsequently, a stringent filter was applied to exclude nuclei with fewer than 500 detected genes or that contained more than 5% of organelle genes.

Unsupervised clustering was performed using Seurat (v4.4.3) [53]. Firstly, quality control was conducted with UMI more than 500 and less than 5000. Then, ‘NormalizeData’ and ‘ScaleData’ functions in Seurat package were used to normalize UMI count matrices, principal component analysis (PCA) was performed to dimension reduction using ‘Run PCA’ function, ‘FindClusters’ and ‘RunUMAP’ were used to clustering and dimensionality reduction. The clustering resolution parameters were set at 0.8 after a series of resolution adjustments.

Data integration among samples was performed by ‘Run Harmony’ to batch effects between samples, and 3D UMAP graph was constructed with ‘RunUMAP’. ‘FindAllMarkers’ was used to identify the cluster-specific genes with parameters min.pct = 0.25 and logfc threshold = 0.25; genes with |log2FC| > 0.25 and *P* value adjust <0.05 would be retained for subsequent analysis. Previously known marker genes from tomato and homologous genes from *Arabidopsis* were used to identify cell type annotation [14, 16, 17].

### Differential expression analysis and GO enrichment

Differential expression analysis was performed on ACC or Torin2 treatment and mock of hooks and hypocotyls by ‘FindMarkers’. Genes with |log2FC| > 0.25 and *P* value adjust <0.05 would be retained for subsequent analysis. To annotate the clusters, we constructed a tomato enrichment package based on clusterProfiler (v4.12.6) and AnnotationForge (v1.40.2) [54, 55]. Terms with *P* value <0.05 would be retained and plotted by ggplot2 (v3.5.2). The ‘AddModuleScore’ was used to calculate the score of specific expressed gene lists, and the score results were plotted by ggplot2 (v3.5.2) [56]. Average expression values for each cell-type were computed by ‘AverageExpression’ function in Seurat. Spearman’s correlation coefficients were calculated and plotted by heatmap (v1.0.12) [57].

### Developmental trajectory analysis

Cell differential was computed by CytoTRACE (v0.3.3) [40], and pseudotime trajectory analysis was performed by Monocle (v2.24.0) [58] on each sample of apical hooks and hypocotyls. Developmental trajectory analysis was performed with the following steps: variable genes were selected with expression in at least three cells and gene expression levels over 0.1 by ‘detectGenes’, ‘DDRTree’ was utilized to perform dimensionality reduction to two dimensions (max_components = 2), cells were ordered along trajectory by ‘orderCells’ and visualized by ‘plot_cell_trajectory’, and branched-dependent genes were computed and clustered by ‘BEAM’ and visualized by ‘plot_genes_branched_heatmap’.

### Gene modules analysis

To cluster genes with similar expression patterns into modules, gene modules were constructed using the R package Monocle 3 (v1.0.0) [59]. Briefly, the data were converted into a cell_data_set (cds) object using ‘new_cell_data_set’ function. The cds was preprocessed using ‘preprocess_cds’ and ‘reduce_dimension’ functions. The gene modules of special expression patterns were grouped using ‘find_gene_modules’ function and visualized using pheatmap.

WGCNA analysis [60] was performed based on the average gene expression levels from each sample with different treatments. The network diagrams were visualized using Cytoscape (v3.10.3) [61].

### TF interaction network

TFs were downloaded on Phytozome (https://phytozome-next.jgi.doe.gov/). The specific expressed TFs were utilized to construct the protein–protein interaction network by STRING (https://string-db.org/). The TF interaction network was constructed by Cytoscape (v3.10.3) [61].

### CRISPR/Cas9-mediated loss of function of JA1

To knock out *JA1* by CRISPR/Cas9, sgRNAs were designed containing 20-bp target sequences specific to the 5′-coding regions of JA1 followed by the NGG protospacer adjacent motif. The designed sgRNAs were cloned into the binary vector pHNRhCas9NG as previously described [62]. To obtain mutant lines, the construct was transformed into tomato by cocultivation of cotyledons derived from 8-day-old seedlings using *Agrobacterium tumefaciens*-mediated transformation (strain GV3101). The homozygous alleles were identified by sequencing of PCR amplicons from their respective DNA extracts from T1 progenies.

### Ethylene triple response assay

Germinated seeds of WT and ja1 mutants were sown on 1/2 MS medium with or without 1 μM ACC and cultured at 25°C under darkness. Root length, hypocotyl length, and hook curvature were measured at 72 h postsowing. For each line, at least 10 seedlings were measured.

## Supplementary Material

Web_Material_uhag044

## Data Availability

The sequencing data generated in this study have been deposited in the China National GeneBank (CNGB) Sequence Archive (CNSA) [63] with the accession number CNP0007766. Scripts used for snRNA-seq data processing in this study are available in GitHub (https://github.com/ctan2020/Tomato-hook-SC).
