# Supplementary material for: Single-nucleus transcriptome profiling unveils cell-type-specific ethylene and TOR signaling in tomato
Source: Hortic Res. 2026 Feb 28;13(5):uhag044. doi: 10.1093/hr/uhag044 (PMC13156461; doi:10.1093/hr/uhag044)
Supplement: Web_Material_uhag044 [file web_material_uhag044.zip › Supplemental Figures.pdf]

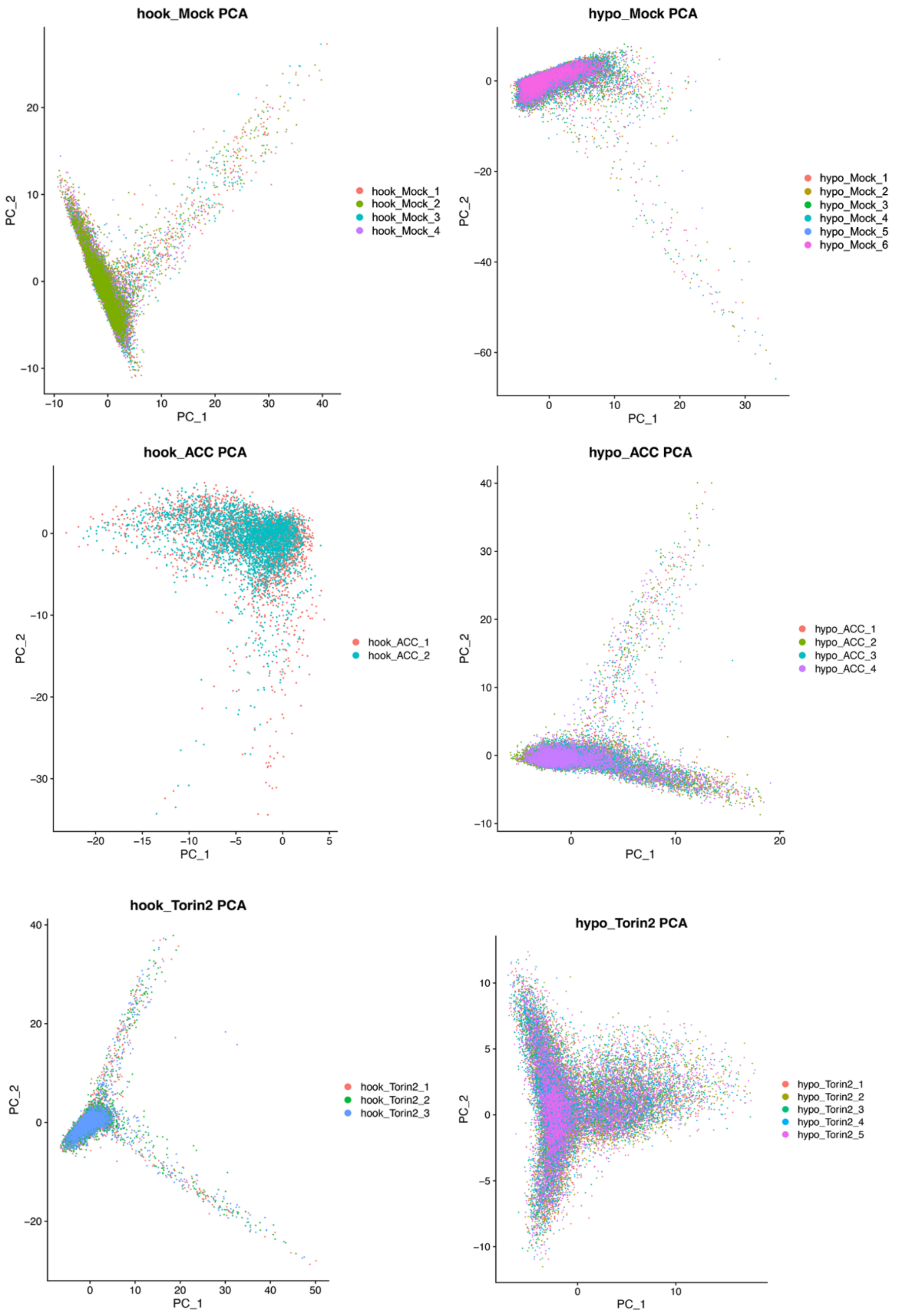


**Figure S1** | PCA analysis of different libraries in each of the six samples.

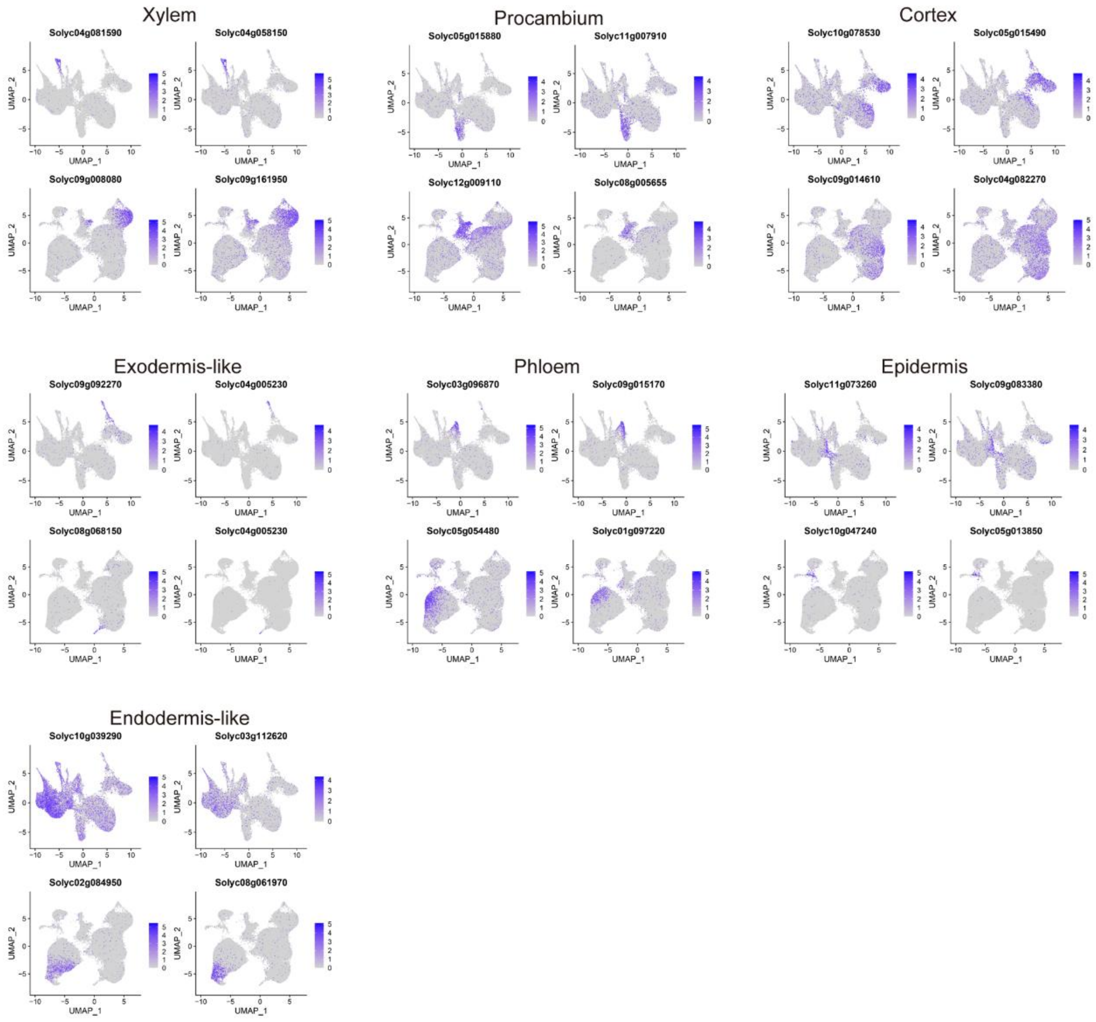


**Figure S2 |** UMAP plot illustrating the spatial distribution of expression of selected marker genes in seven cell types of apical hooks and hypocotyls.

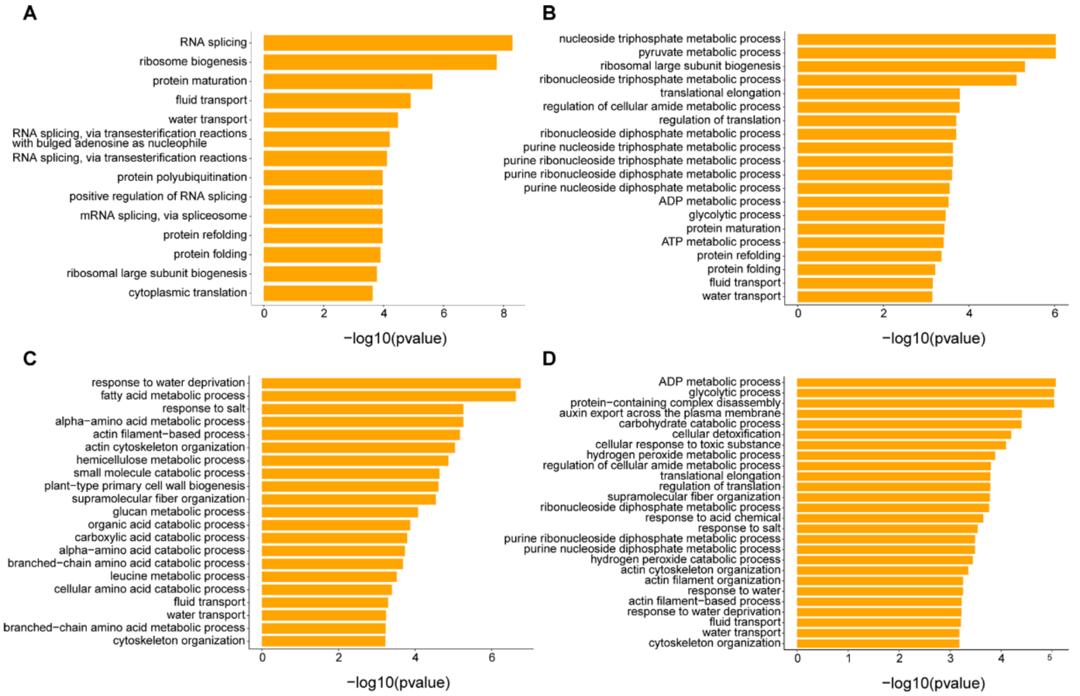


**Figure S3** | GO enrichment analyses of the 1,456 DEGs in apical hooks with ACC treatment (A), the 529 DEGs in apical hooks with Torin2 treatment (B), the 1104 DEGs in hypocotyls with ACC treatment (C), and the 1517 DEGs in hypocotyls with Torin2 treatment (D).

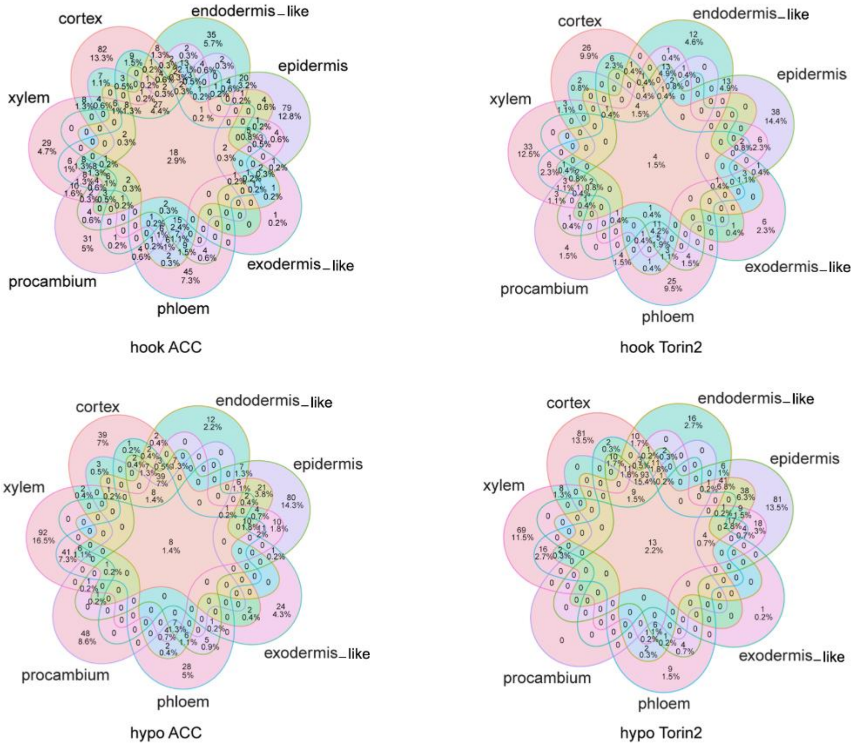


**Figure S4** | Venn diagram showing numbers of overlapping and specific DEGs between seven cell types of apical hooks and hypocotyls with ACC or Torin2 treatment.

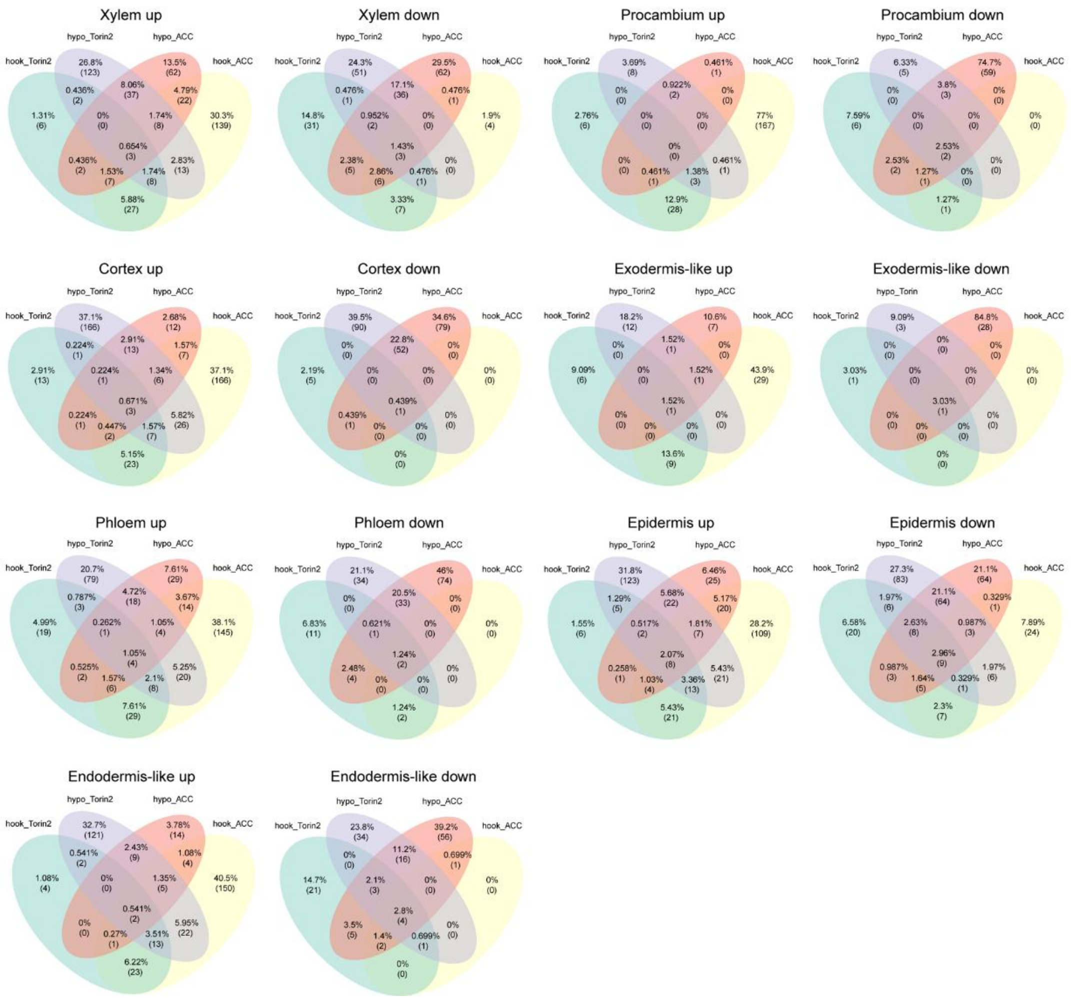


**Figure S5** | Venn diagram showing DEGs of individual cell type that are specifically or commonly regulated by ACC or Torin2 treatment in apical hooks and hypocotyls.

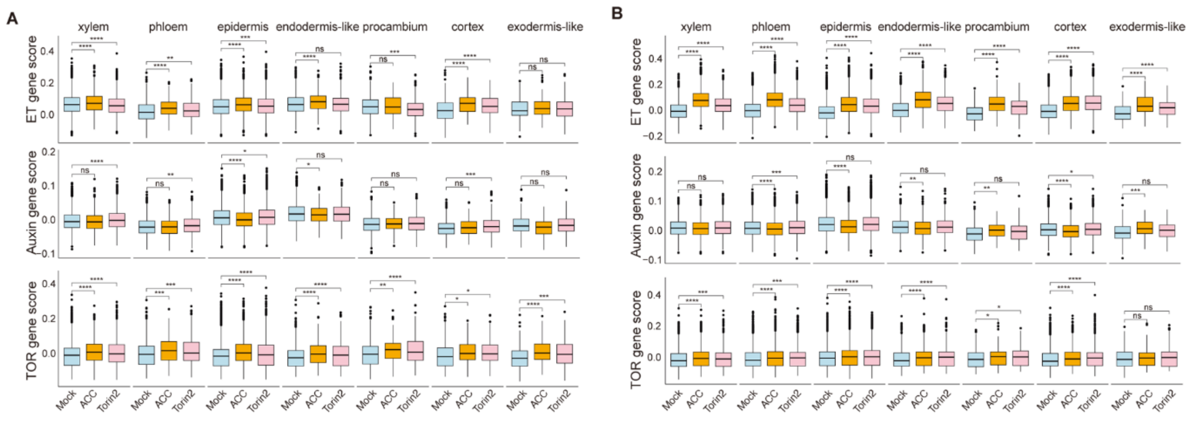


**Figure S6** | Average expression score of ethylene, auxin, and TOR pathway genes upon ACC/Torin2 treatments in apical hooks (A) and hypocotyls (B).

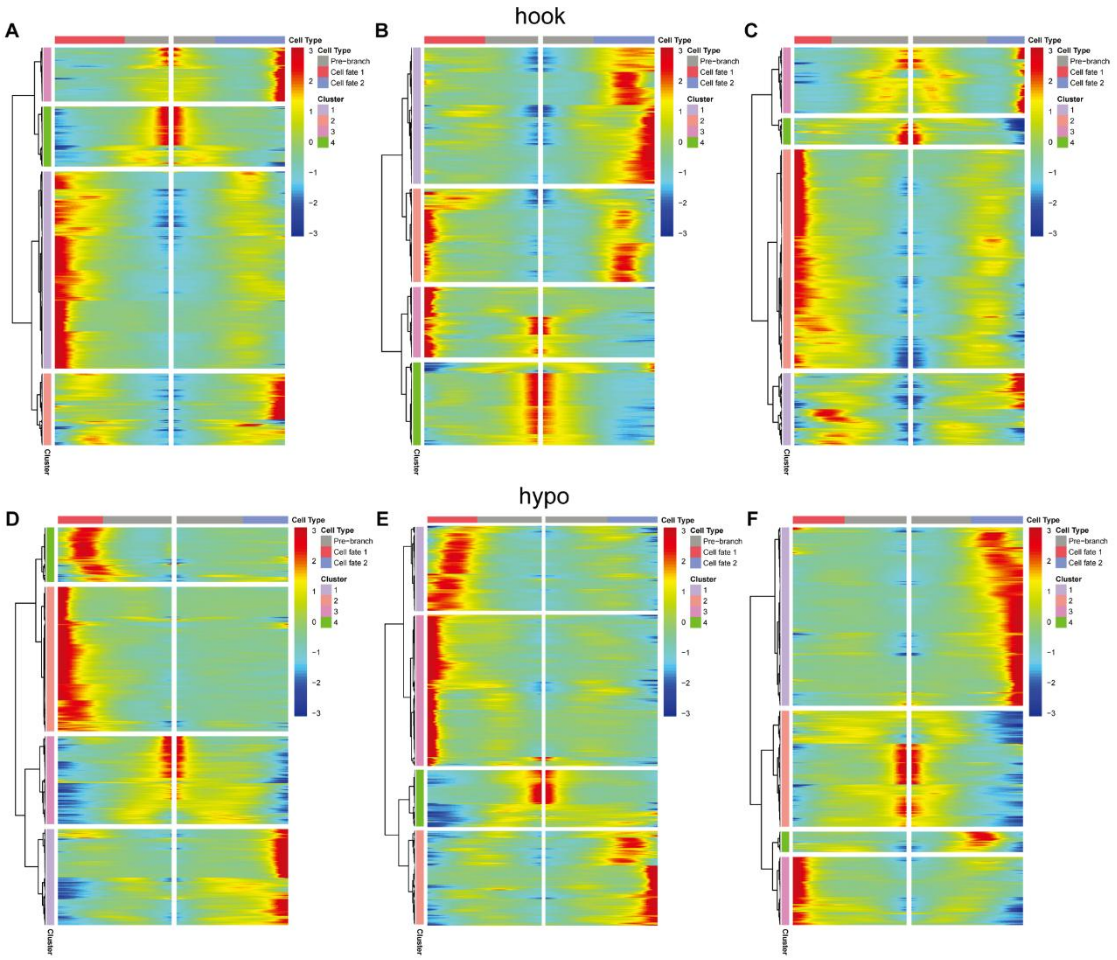


**Figure S7** | Heatmap displaying highly significant branch-specific expression patterns along the pseudo-time trajectories in apical hooks under Mock (A), ACC treatment (B), Torin2 treatment (C), and hypocotyls under Mock (D), ACC treatment (E), and Torin2 treatment (F).

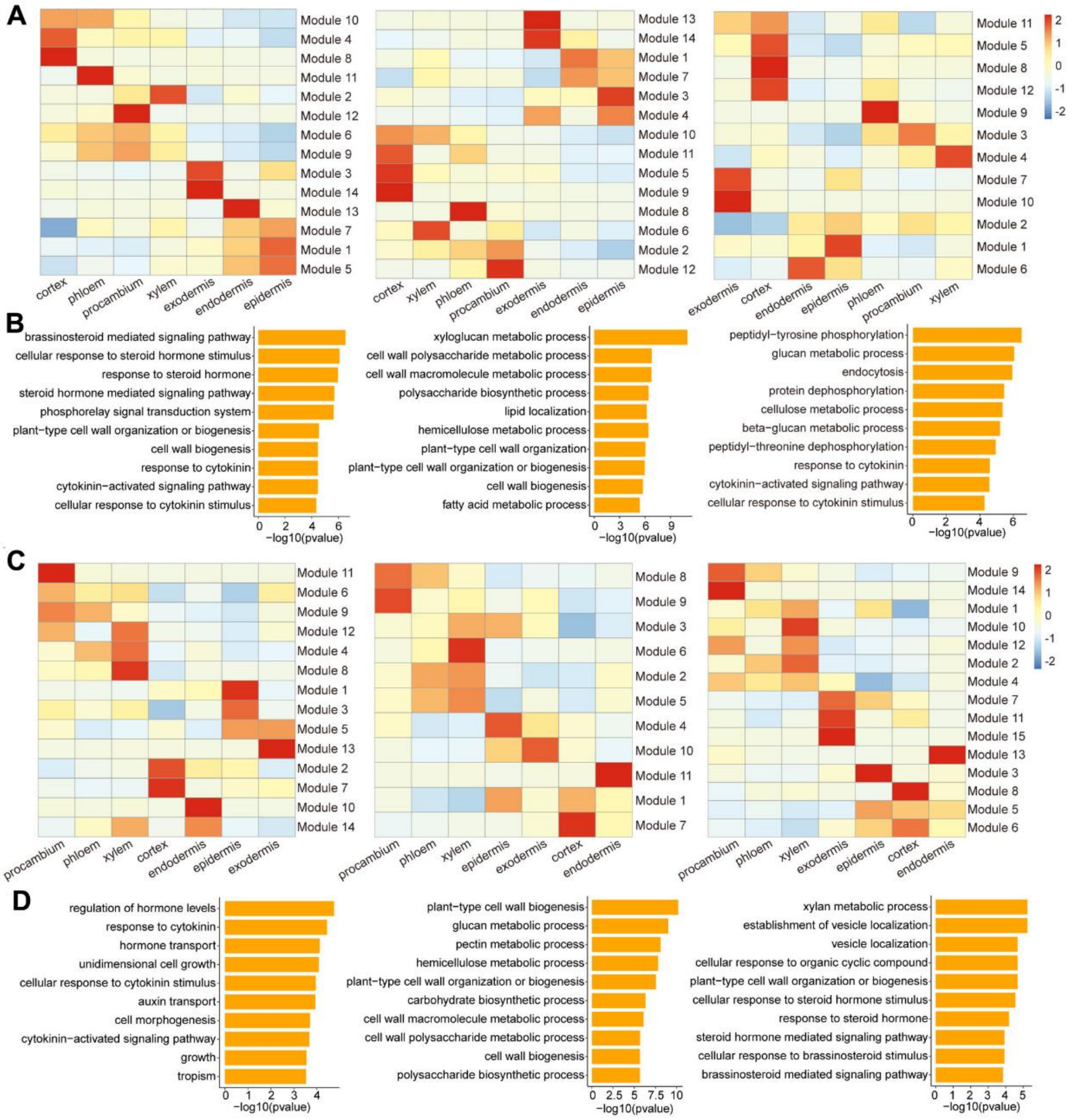


**Figure S8** | Identification of the gene modules associated with epidermis and the corresponding GO term analysis in apical hooks (A and B) and and hypocotyls (C and D).

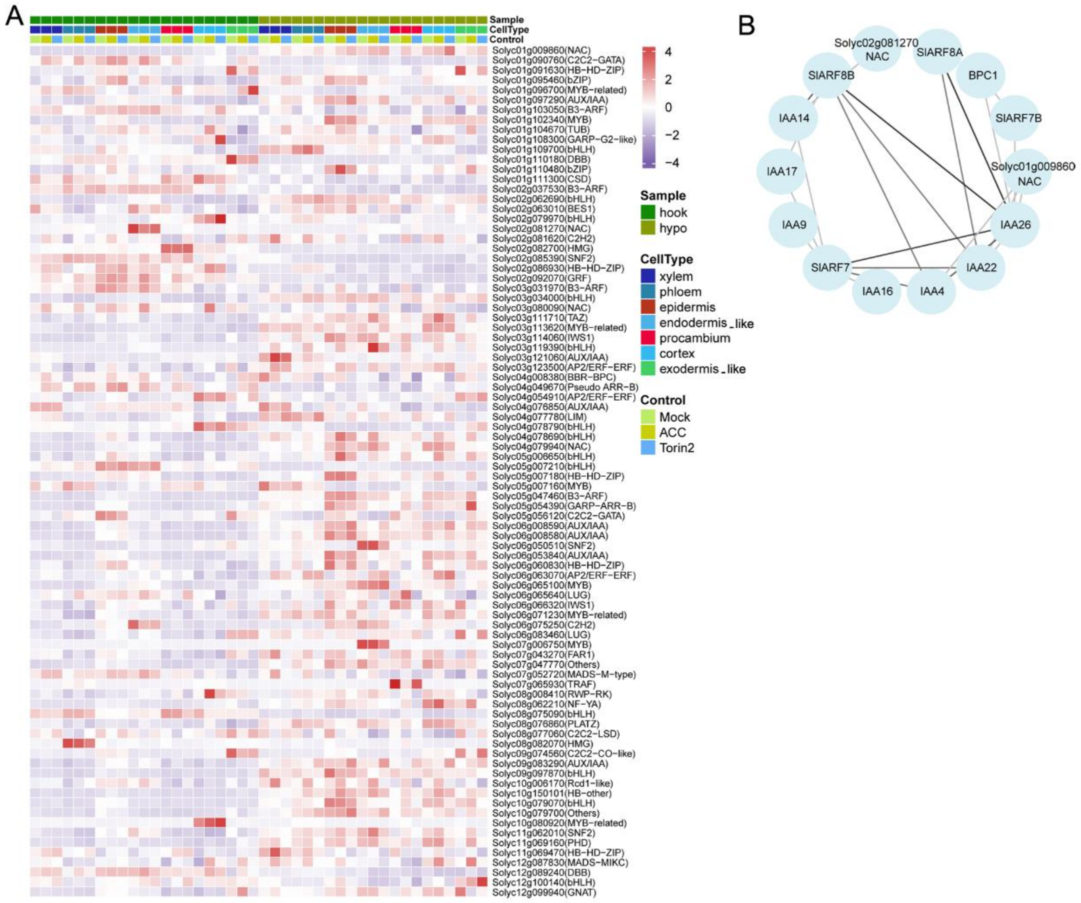


**Figure S9 |** Heatmap showing the differentially expressed TF genes in each of the seven cell types responding to ACC or Torin2 treatment (A) and the putative protein–protein interaction network of 14 differentially expressed TF genes (B).

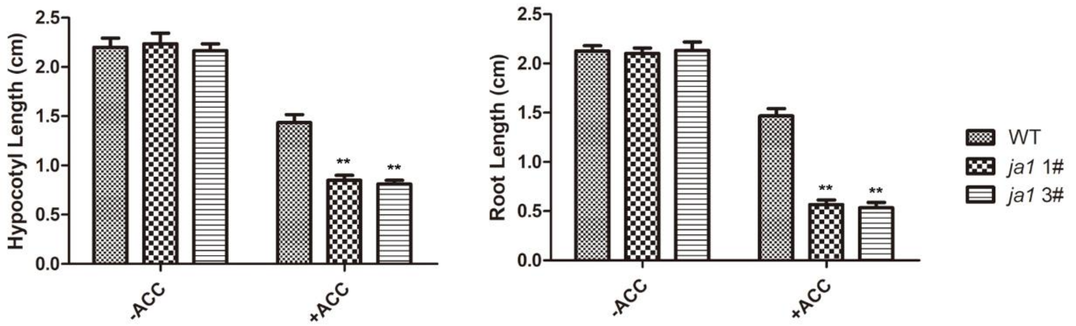


**Figure S10** | Statistics of hypocotyl and root length of WT and two *ja1* mutant alleles. The error bars indicate the SDs (n = 10). **P < 0.01, Student's t test.
